# Genetic and Epigenetic Changes Are Rapid Responses of the Genome to the Newly Synthesized Autotetraploid *Carassius auratus*

**DOI:** 10.3389/fgene.2020.576260

**Published:** 2021-01-07

**Authors:** Chongqing Wang, Yuwei Zhou, Huan Qin, Chun Zhao, Li Yang, Tingting Yu, Yuxin Zhang, Tao Xu, Qinbo Qin, Shaojun Liu

**Affiliations:** ^1^State Key Laboratory of Developmental Biology of Freshwater Fish, Engineering Research Center of Polyploid Fish Reproduction and Breeding of the State Education Ministry, College of Life Sciences, Hunan Normal University, Changsha, China; ^2^Hunan Normal University, Changsha, China

**Keywords:** polyploidy fish, genetic, epigenetic alternation, DNA methylation, full-length transcriptome

## Abstract

Whole genome duplication events have occurred frequently during the course of vertebrate evolution. To better understand the influence of polyploidization on the fish genome, we herein used the autotetraploid *Carassius auratus* (4n = 200, RRRR) (4nRR) resulting from the whole genome duplication of *Carassius auratus* (2n = 100, RR) (RCC) to explore the genomic and epigenetic alterations after polyploidization. We subsequently performed analyses of full-length transcriptome dataset, amplified fragment length polymorphism (AFLP) and methylation sensitive amplification polymorphism (MSAP) on 4nRR and RCC. By matching the results of 4nRR and RCC isoforms with reference genome in full-length transcriptome dataset, 649 and 1,971 novel genes were found in the RCC and 4nRR full-length geneset, respectively. Compared to *Carassius auratus* and *Megalobrama amblycephala*, 4nRR presented 3,661 unexpressed genes and 2,743 expressed genes. Furthermore, GO enrichment analysis of expressed genes in 4nRR revealed that they were enriched in meiosis I, whereas KEGG enrichment analysis displayed that they were mainly enriched in proteasome. Using AFLP analysis, we noted that 32.61% of RCC fragments had disappeared, while 32.79% of new bands were uncovered in 4nRR. Concerning DNA methylation, 4nRR exhibited a lower level of global DNA methylation than RCC. Additionally, 60.31% of methylation patterns in 4nRR were altered compared to RCC. These observations indicated that transcriptome alterations, genomic changes and regulation of DNA methylation levels and patterns had occurred in the newly established autotetraploid genomes, suggesting that genetic and epigenetic alterations were influenced by autotetraploidization. In summary, this study provides valuable novel insights into vertebrate genome evolution and generates relevant information for fish breeding.

## Introduction

Polyploidy plays a vital role in the evolution of the vertebrates (Peer et al., 2017). According to Li et al. (2010), the majority of vertebrates are generated from an ancestor via two or three rounds of whole-genome duplication. Generally, autopolyploids and allopolyploids are distinguished based on the chromosomal composition and manner of formation (Sumitomo et al., 2019). The former carries multiple similar chromosome sets (e.g., AAAA) where each chromosome contains two or more potential partners resulting in the formation of multivalent pairing during meiosis. On the other hand, the latter integrates two or more different species genomes (e.g., AABB), which undergo bivalent pairing because meiotic pairing occurs between homologous chromosomes (Sumitomo et al., 2019). Traditionally, autopolyploids are considered to undergo several evolutionary problems compared to allopolyploids because multivalent pairing and unstable meiosis can cause reduced fertility. Numerous reports have widely demonstrated the consequences of genome duplication in allopolyploids (Skalická et al., 2005; Xiong et al., 2011). However, there is a paucity of available information on autopolyploids. Recent accumulating evidence suggests that autopolyploidy might have a greater impact on species diversification and evolution than previously thought (Barker et al., 2016).

Polyploidy organisms contain multiple sets of genetic material. A study by Ramsey and Schemske (2002) reported that increased genome dosage in polyploids causes strongly increased frequency of instabilities of the genome, imbalances of chromosomes, incompatibilities of regulation and loss of fertility. Therefore, to establish a new equilibrium in the two or more genomes, new polyploids must experience strong genome restructuring and wide reorganization of gene expression after genomic merging and doubling (Levy and Feldman, 2004). It has been established that various genomic changes exhibit a positive effect on diploid-like chromosome pairing (Moore, 2002). However, despite advances in our understanding of genomic changes associated with the evolutionary success of polyploidy, epigenetic underlying mechanisms remain elusive (Lee and Chen, 2001; Chen et al., 2008). Notably, DNA methylation is a common epigenetic phenomenon that may reprogram patterns of gene expression and developmental patterns of neopolyploids. Thus, epigenetic mechanisms assist in the evolution of a polyploid (Chen, 2007).

Genetic and epigenetic changes enhance the adaptive evolution of newly formed polyploid species, causing important phenotypic consequences and appearance (Comai et al., 2000; Schranz and Osborn, 2000, 2004; Madlung et al., 2002). Noticeably, polyploid species and individuals are common in plants but rarely reported in vertebrates. As a result, studies of genomic and epigenetic modifications associated with polyploid have been primarily focused mostly on polyploid plants (Ozkan et al., 2001; Salmon et al., 2005), but little attention has been paid to polyploid vertebrates. In our previous study, we successfully obtained fertile autotetraploid *Carassius auratus* (4n = 200, RRRR) (abbreviated as 4nRR) derived from distant hybridization of *Carassius auratus* (2n = 100, RR) (abbreviated as RCC) (♀) × *Megalobrama amblycephala* (2n = 48, BB) (abbreviated as BSB) (♂) (Qin et al., 2014). This autotetraploid exhibited normal chromosomal behavior during meiosis and maintained the formation of the autotetraploid line (F_1_–F_10_) (Qin et al., 2019a). Besides, it offers a unique model for investigating genetic and epigenetic alterations of autopolyploids in vertebrates. In this paper, we conducted the full-length transcriptome dataset, AFLP and MSAP analyses to reveal genome-wide changes in genetic and DNA methylation in the 4nRR genome. This experiment allowed extending the knowledge about the role polyploidization plays in genetic and epigenetic alterations in fish, and is effective in clearly illustrating the genome evolution of vertebrates.

## Materials and Methods

### Ethics Statement

All experimental procedures on fish were reviewed and approved by the Institute of Experimental Animals, Hunan Province, China. Fish was deeply anesthetized with 100 mg/L MS-222 (Sigma-Aldrich, St Louis, MO, United States) before blood and liver tissue surgically collection.

### Animals Preparation

Ten individuals each of females RCC and 4nRR were acquired from the State Key Laboratory of Developmental Biology of Freshwater Fish, Hunan Normal University, China at 12 months of age. During the reproductive seasons (April) of 2019, RCC of RCC (♀) × RCC (♂) and 4nRR of 4nRR (♀) × 4nRR (♂) were produced. All fish were cultured in open pools (0.067 ha) in which its pH was (7.0–8.5), water temperature (22–24°C), dissolved oxygen content (5.0–8.0 mg/L) and adequate forage. The ploidy types of each sample were determined using a flow cytometer (BD Biosciences, San Jose, CA, United States). Using heparinized syringes, we collected red blood cells from the caudal vein of each fish. Then, blood samples were resuspended in staining solution (NIM-DAPI 731085) (NPE systems, Pembroke Pines, FL, United States) for 10 min. We compared the total DNA content of each fish was compared with that of RCC (Xiao et al., 2011).

### Fluorescence *in situ* Hybridization (FISH)

For this experiment, chromosome preparations were obtained from peripheral blood of 10 RCC and 10 4nRR as described by Luo et al. (2011). We used the primers 5’-TTCGAAAAGAGAGAATAATCTA-3’ and 5’-AACTCGTCTAAACCCG AACTA-3’ to amplify the FISH probes of the 5S gene using PCR. The FISH probes were generated by the use of Dig-11-dUTP labeling (using a nick translation kit; Roche, Life Science, Penzberg, Upper Bavaria, Germany) of the purification of the PCR product. We then performed the FISH experiment following the method described by He et al. (2012). Finally, metaphase chromosome spreads of 10 RCC and 10 4nRR were analyzed using FISH.

### Tissue Collection and RNA Isolation

In this subsection, we carefully dissected the liver tissue from fish after anaesthetization. The RNA in liver tissue was isolated according to a standard TRIzol protocol (Invitrogen, United States), and consequently qualified and quantified using gel electrophoresis and 2100 Bioanalyzer system (Agilent, United States). Lastly, equal amounts of the RNA from 10 RCC (or 10 4nRR) were mixed as one sample for the subsequent analysis.

### PacBio cDNA Library Preparation and PacBio Sequencing

Synthesis of cDNA was conducted using the SMARTerTM PCR cDNA Synthesis Kit (Clontech, Mountain View, CA, United States). The amplified library was size-selected using BluePippin^TM^ Size Selection System (Sage Science, Beverly, MA, United States) after one round of PCR amplification. A library of 0.5–6 kb was constructed and sequenced using two cells on a PacBio Sequel platform (PacBio, CA, United States).

### PacBio Sequencing Result Processing

We performed raw data analysis using SMRTlink 5.0 software^1^. Circular consensus sequences (CCS) were processed with the SMRT Analysis Software (Pacific Biosciences, United States) and then classified the ROIs into full-length (FL) and non-full-length (nFL) reads. We employed iterative clustering for error correction (ICE) was employed to obtain consensus reads from FL reads. Then, FL consensus reads from ICE were used to polish using Quiver software to obtain high quality isoforms (HQ isoforms) (accuracy ≥ 99%) and low quality isoforms (LQ isoforms) (accuracy < 99%).

### Comparison of HQ and LQ Isoforms With the Reference Genome

HQ and LQ isoforms from RCC and 4nRR were mapped against the RCC reference genome sequence^2^ using GMAP (Wu and Watanabe, 2005), and generated the sam format of alignments. Search for fusion gene from the alignment results was performed using the fusion_finder.py (in cDNA_Cupcake software). After deleting fusion genes, the collapse_isoforms_by_sam.py (in cDNA_Cupcake software) was used to remove redundant sequences in the matching results with min-identity of 0.95 and min-coverage of 0.85. Then MatchAnnot software^3^ was used to annotate matched isoforms. In this respect, using LncTar software, we performed the lncRNA prediction of unannotated isoforms (Li et al., 2015). If the isoform was not predicted to be lncRNAs, then it was considered as the likely novel gene. Subsequently, a similarity search with novel genes was conducted as follows: Blast^4^ with evalue < 0.00001, against the COG database^5^, KEGG database^6^, NR (non-redundant) database^7^, Swiss-Prot protein database^8^, and GO database^9^. Consequently, RCC and 4nRR annotated isoforms can be obtained. We screened only those isoforms that were present in the 4nRR using the Venny 1.0 software^10^. Sequences corresponding to the isoforms were then aligned to the BSB reference genome (unpublished) with blastn (version 2.9.0). After removing the matched isoforms, the remaining isoforms were identified as the 4nRR-specific isoforms (expressed genes), whereas the RCC-specific isoforms (the isoforms that only were present in the RCC) were identified as unexpressed genes in 4nRR. To reveal the functions of the expressed genes in 4nRR, we executed Gene Ontology (GO) and Kyoto Encyclopedia of Genes and Genomes (KEGG) enrichment analysis with clusterProfiler R package (version 3.14.0) to annotate them.

### AFLP Analysis

Genomic DNA was isolated from peripheral blood of 5 RCC and 5 4nRR using Rapid Blood Genomic DNA Isolation Kit (Sangon Biotech). We subsequently performed the AFLP experiment and analysis based on the method elucidated by Qin et al. (2020). According to the presence or absence of bands, AFLP data for PCR products electrophoresis were converted into a binary matrix of 1 and 0. Afterward, we compared the banding patterns of the amplification products were compared between RCC and 4nRR based on the AFLP data, where bands were classified into three types (Table 1).

**TABLE 1:** AFLP fragments that were observed in *Carassius auratus* (RCC) and in autotetraploid *Carassius auratus* (4nRR) generated by *Pst*I–*Mse*I digestions (eight selective primer combinations).

Type	RCC	4nRR	Number of sites
A(special fragments in 4nRR)	0	1	181
B(special fragments in RCC)	1	0	180
C(common fragments in RCC and 4nRR)	1	1	191

*1, the place with the band is replaced by 1; 0, the place without the band is replaced with 0.*

### MSAP Analysis

Here, we sampled 10 RCC and 10 4nRR for isolation of genomic DNA. We performed the MSAP experiment and analysis as per the method described by Qin et al. (2020). Based on the presence or absence of bands, MSAP data for PCR product electrophoresis can be considered as a matrix of 1 and 0. The banding types of the amplification products of each type of fish (RCC and 4nRR) were characterized into three types, including unmethylated, hemi-methylated and fully methylated (Table 2). Further analyses of DNA methylation patterns within RCC and 4nRR, and methylation patterns were divided into three types (Table 3).

**TABLE 2:** MSAP fragments that were observed in *Carassius auratus* (RCC) and in autotetraploid *Carassius auratus* (4nRR) generated by *Eco*RI–*Hpa*II/*Eco*RI–*Msp*I digestions (twenty selective primer combinations).

Type	*Hpa*II	*Msp*I	RCC	4nRR
Hemimethylation	1	0	120(40.00%)	117(28.61%)
Fully methylation	0	1	43(14.33%)	87(21.27%)
Non-methylation	1	1	137(55.67%)	205(46.12%)
Total methylation			163(54.33%)	204(49.88%)

*1, the place with the band is replaced by 1; 0, the place without the band is replaced with 0.*

**TABLE 3:** Methylation patterns that were observed in *Carassius auratus* (RCC) and in autotetraploid *Carassius auratus* (4nRR) generated by *Eco*RI–*Hpa*II/*Eco*RI–*Msp*I digestions (twenty selective primer combinations).

Methylation patterns	4nRR		RCC		Number of sites	Total
	*Hpa*II	*Msp*I	*Hpa*II	*Msp*I		
A (present in both RCC and 4nRR)	0	1	0	1	7	
	1	0	1	0	15	39.69%
	1	1	1	1	132	
					154	
B(demethylation)	1	1	1	0	22	
	1	1	0	1	17	24.26%
	1	1	0	0	17	
	1	0	0	0	25	
	0	1	0	0	13	
					94	
C(hypermethylation)	1	0	1	1	38	
	0	1	1	1	24	36.05%
	0	0	1	1	35	
	0	0	1	0	23	
	0	0	0	1	20	
					140	

*1, the place with the band is replaced by 1; 0, the place without the band is replaced with 0.*

## Results

### Ploidy Identification

**FIGURE 1:**
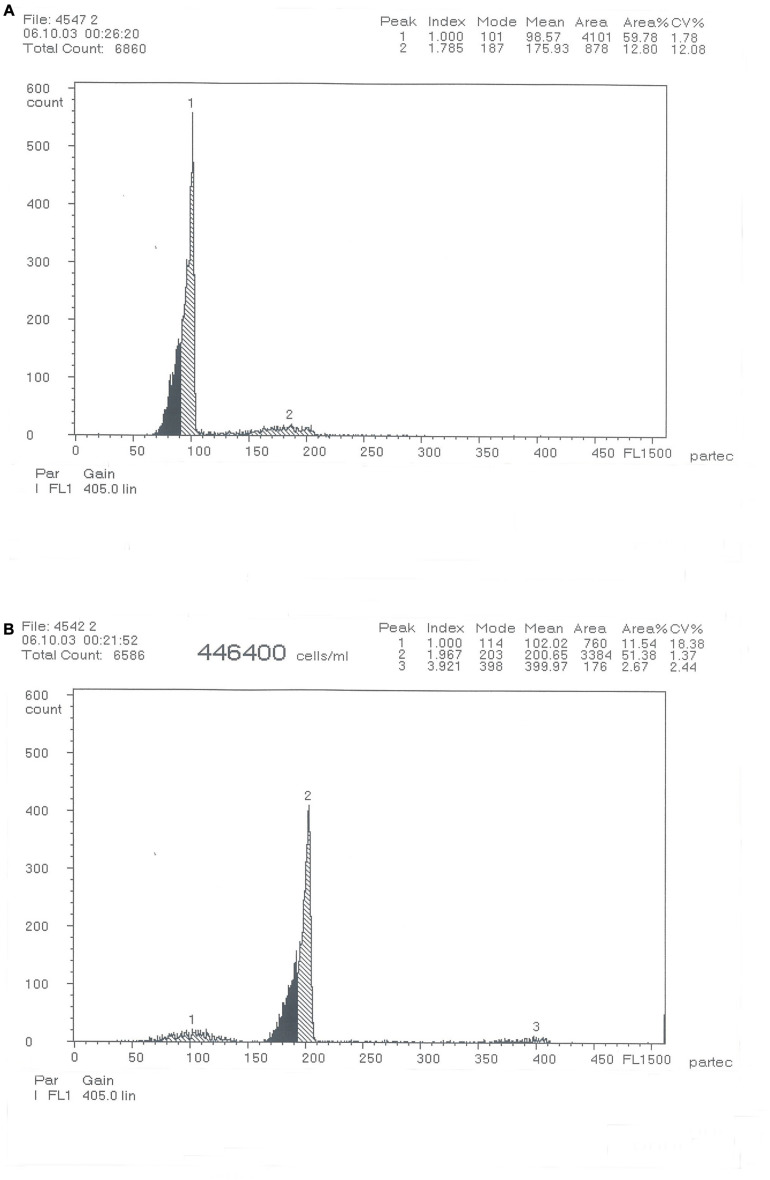
DNA-content flow-cytometrical histograms of *Carassius auratus* red var. (RCC) **(A)** and Autotetraploid *Carassius auratus* (4nRR) **(B)**.

The distribution of DNA content measured using flow cytometry is presented in Figure 1. The DNA content ratio in individual blood cells of RCC and 4nRR forms was 1:2.

### Fluorescence *in situ* Hybridization (FISH)

**FIGURE 2:**
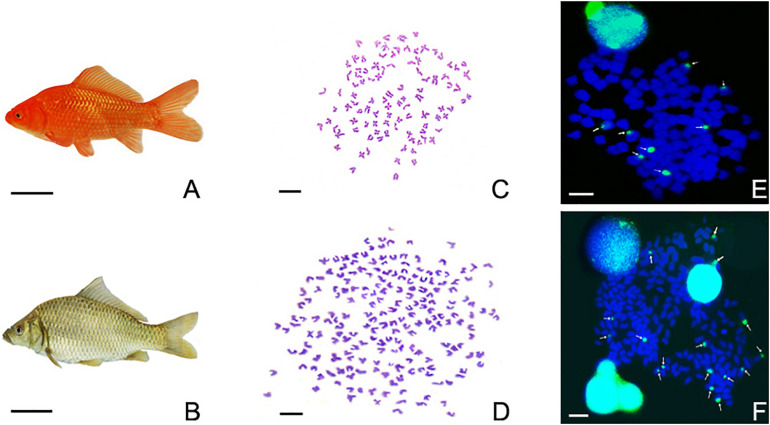
The morphological appearances, chromosome metaphase spreads, and examination of FISH signals in RCC, and 4nRR. **(A)** *Carassius auratus* red var. (RCC). **(B)** Autotetraploid *Carassius auratus* (4nRR). The bar in **(A,B)** is representative of 1 cm. **(C)** The 100 chromosome of RCC. **(D)** The 200 chromosome of 4nRR. **(E)** There were eight 5S gene loci in RCC (indicated by white arrows). **(F)** There were sixteen 5S gene loci in 4nRR (indicated by white arrows). The bar in **(C–F)** is representative of 3 μm.

As shown in Figure 2, the appearance of the RCC (Figure 2A) and 4nRR (Figure 2B) was different. In particular, in RCC, metaphase spreads showed 100 chromosomes (Figure 2C), while in 4nRR, they revealed 200 chromosomes (Figure 2D). Moreover, the metaphase chromosomes of RCC and 4nRR were hybridized to the 5S gene probe (GenBank Accession No. GQ485557). Subsequently, FISH observations results demonstrated that the chromosomal metaphases of RCC possessed eight 5S gene loci (Figure 2E), whereas 4nRR exhibited sixteen 5S gene loci in the chromosomal metaphases (Figure 2F), which was two times as much as RCC. This finding suggested that 4nRR possessed four sets of RCC-derived chromosomes.

### Overview of Transcripts From PacBio Sequencing in RCC and 4nRR

In RCC, we successfully extracted 162,656 reads of inserts (ROIs) from 3,336,880 polymerase reads, and the mean insert read length and number of passes were 1,896 bp and 18.53, respectively. Overall, 152,624 FL non-chimeric transcripts were obtained from the ROIs. Additionally, in 4nRR, we successfully isolated 130,984 ROIs from 3,693,315 polymerase reads, and the average insert read length and number of passes were 1,623 bp and 26.21, respectively. Subsequently, 123,859 FL non-chimeric transcripts were derived from the ROIs (Table 4).

**TABLE 4:** Summary of reads of sequencing.

Sample	Number of polymerase reads	Number of reads of insert	Mean read length of Insert	Mean number of passes	Number of full-length non-chimeric reads
RCC	3,336,880	162,656	1,896	18.53	152,624
4nRR	3,693,315	130,984	1,623	26.21	123,859

*RCC, Carassius auratus; 4nRR, autotetraploid Carassius auratus.*

### Analysis of Transcripts Between RCC and 4nRR

**FIGURE 3:**
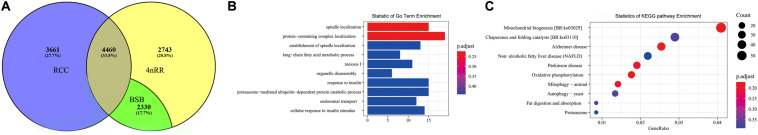
Transcriptome analysis. **(A)** Annotation genes of *Carassius auratus* red var. (RCC), *Megalobrama amblycephala* (BSB), and autotetraploid *Carassius auratus* (4nRR). **(B)** The enriched molecular functions of acquired genes in 4nRR. **(C)** The enriched pathways of acquired genes in 4nRR.

After removal of the fusion genes, 97,149 (accounting for 97.14%) of the remaining 100,009 isoforms in RCC were identified by matching the RCC reference genome. Overall, we obtained 47,352 unique isoforms after preprocessing of filtered and elimination of redundancy with 97,149 matching isoforms. In total, 14,198 of the 47,352 unique isoforms matched the reference genome annotation sequences, thus mapping 8,121 annotation genes in the reference genome. Additionally, in 4nRR, we obtained 87,909 (accounting for 99.50%) of the remaining 88,347 isoforms by matching the RCC reference genome after removal of the fusion genes. Then, after preprocessing of filtered and elimination of redundancy with 87,909 matching isoforms, we annotated a total of 21,834 unique isoforms. Notably, 17,528 of the 21,834 unique isoforms matched the reference genome annotation sequences, hence mapping 9,533 annotation genes in the reference genome. In comparison, 4nRR possessed 2,743 expressed genes and 3,661 unexpressed genes than RCC and BSB genomes (Figure 3A). Thereafter, we performed functional enrichment analysis was performed on the expressed genes. We found that GO analysis of the expressed genes mainly involved protein-containing complex localization, spindle localization, response to insulin and meiosis I (Figure 3B). In addition, the KEGG pathway annotation enabled us to assign 1,416 genes to 378 pathways. Interestingly, pathway enrichment analysis revealed some enriched pathways, namely, mitochondrial biogenesis, chaperones, and folding catalysts, Alzheimer disease and proteasome (Figure 3C). Moreover, we observed 649 and 1,971 novel genes were observed in the RCC and 4nRR full-length geneset, respectively (Supplementary Table 1). By performing Blast analysis against five public databases (COG, KEGG, NR, Swiss-Prot, and GO) with a threshold of 10^–5^, we annotated 345 novel genes in RCC. Further, our results revealed that 296 (45.61%) novel genes were matched to the Swiss-Prot database, whereas the lowest number of novel genes [101 (15.56%)] were annotated against the NR database (Table 5). In 4nRR, 1,065 novel genes were annotated, while 453 (22.98%) novel genes were matched to the GO database. The highest number of novel genes [1,059 (53.73%)] were annotated against the NR (non-redundant) database (Table 5).

**TABLE 5:** Annotation of novel genes in different databases in *Carassius auratus* (RCC) and autotetraploid *Carassius auratus* (4nRR).

Database	COG		KEGG		NR		Swiss-Prot		GO		Total annotated	
	RCC	4nRR	RCC	4nRR	RCC	4nRR	RCC	4nRR	RCC	4nRR	RCC	4nRR
Gene_number	248	636	130	470	101	1059	296	789	128	453	345	1065
Annotation_Ratio(%)	38.21	32.27	20.03	23.85	15.56	53.73	45.61	40.03	19.72	22.98	53.16	54.03

*COG, Clusters of Orthologous Groups; KEGG, Kyoto Encyclopedia of Genes and Genomes; NR, non-redundant protein sequence; Swiss-Prot, Swiss-Prot protein; GO, Gene Ontology.*

### AFLP Profiling

**FIGURE 4:**
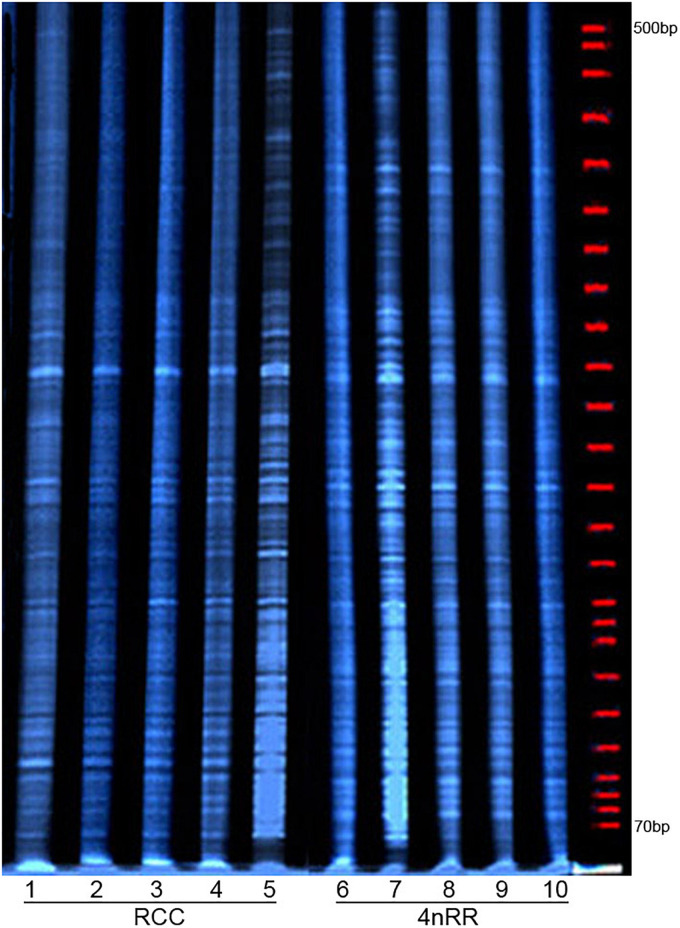
Examples of an AFLP analysis. Numbers 1–5, and 6–10 represent the different individuals of *Carassius auratus* red var. (RCC), and autotetraploid *Carassius auratus* (4nRR), respectively.

To amplify samples in AFLP analysis, we used eight primer combinations as depicted in Supplementary Table 2. These primers produced bands that were incorporated into subsequent data analysis (e.g., Figure 4). In total, we scored and analyzed 552 bands (Table 1). In particular, 181 (32.79%) bands were specific to 4nRR (A-type), 180 (32.61%) bands specific to RCC (B-type), and eventually, 191 (34.60%) bands appeared in both RCC and 4nRR (C-type), demonstrating that 65.40% of the bands were different between RCC and 4nRR. In summary, these findings imply that 4nRR has undergone genomic structural changes in the process of polyploidization.

### Genomic DNA Methylation Levels

Here, twenty primers (Supplementary Table 2) for MSAP analysis produced a total of 704 bands. Of which, 300 bands were detected in the RCC, while 409 were recorded in the 4nRR genome. The rates of total methylation of RCC and 4nRR were 54.33 and 49.88%, respectively (Table 2). These results uncovered that RCC has 163 methylation sites, of which 14.33% (43/300) were fully methylated sites, whereas 40.00% (120/300) were hemimethylated. We further scored 204 methylated sites in 4nRR. Among them, 21.27% (87/409) were fully methylated sites, while 28.61% (117/409) were hemimethylated sites. Collectively, these outcomes demonstrate that the DNA methylation level was lower in 4nRR compared to RCC.

### Methylation Pattern Classification

**FIGURE 5:**
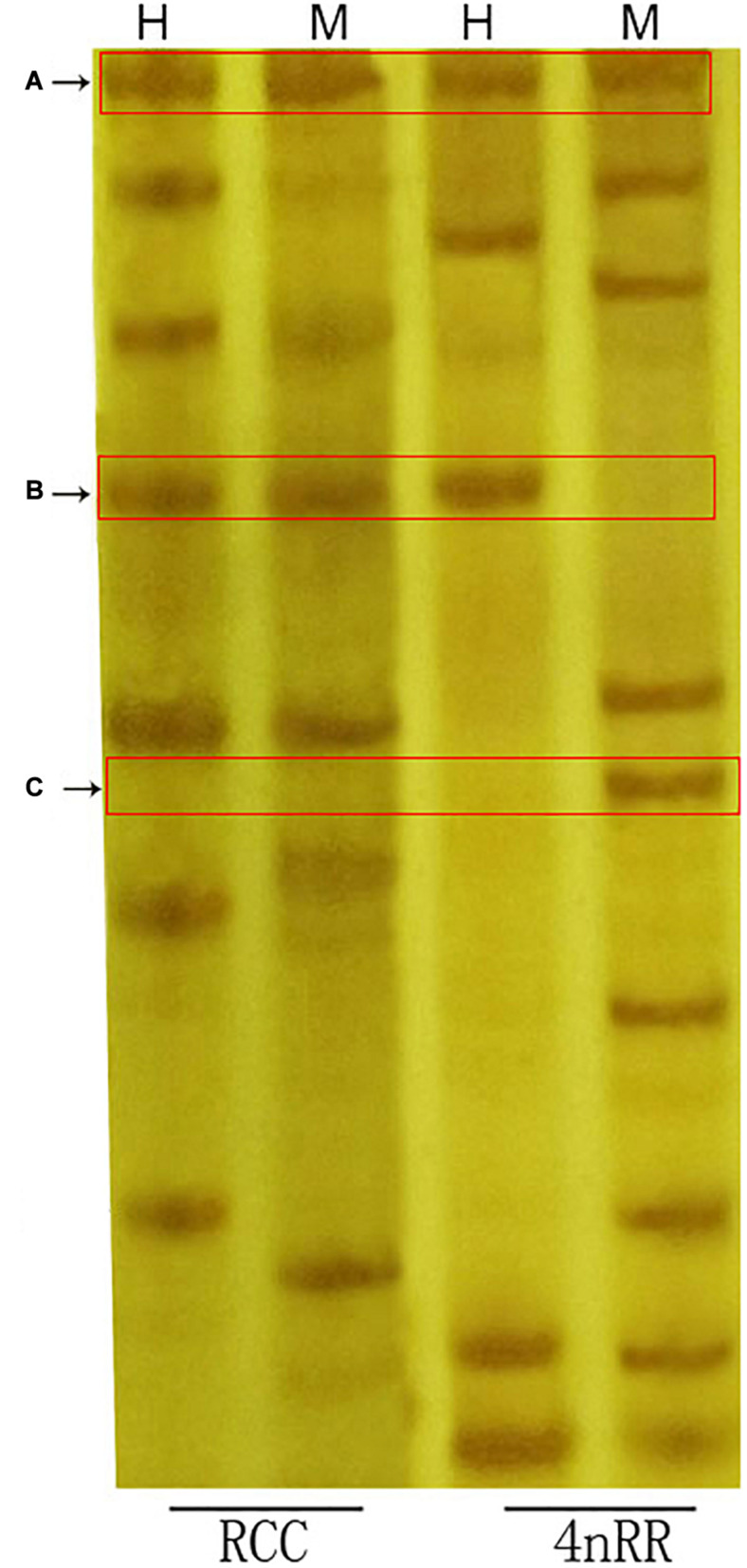
Examples of MSAP analysis. Parts **(A–C)** represent bands corresponding to the methylation types with these labels in Table 3. H, sample digested with *Eco*RI*/Hpa*II; M, sample digested with *Eco*RI/*Msp*I.

We herein used three types (A-C) of DNA methylation patterns were used in the amplified bands of 4nRR and RCC (Table 3 and Figure 5). As shown in Table 3, we observed that 39.69% of the methylation patterns were type A, indicating that the methylation pattern did not change in the 4nRR compared to the RCC. However, the rest of the patterns (60.31%) showed that the methylation sites were altered between 4nRR and RCC. Put another way, 24.26% of the sites showed a decrease in methylation levels (hypomethylation) (B-type), whereas 36.05% of the sites were assigned to hypermethylation (an increase in methylation levels) (C-type). Taken together, these findings implied that many modifications occur in polyploidization methylation patterns.

## Discussion

Allopolyploidy is not uncommon in fish (Zhou and Gui, 2017), which is accompanied by massive DNA changes as well as alterations in DNA methylation that may influence gene expression (Paun et al., 2007). These changes may be in response to polyploidy and hybridization. However, the genetic and epigenetic consequences of autotetraploid remain enigmatic. Therefore, in this present investigation, we analyzed autotetraploid *Carassius auratus* in order to explore the variation of the genomic and epigenetic after genome doubling.

### Genetics Changes in Autotetraploid *Carassius auratus*

Recent genomic studies have established that genomic variations such as *Hox* gene variation, chromosomal rearrangement, and rDNA sequence changes usually occur in the newly established allopolyploid genome due to incompatibility between homologous chromosomes (Qin et al., 2010, 2016; Liu et al., 2017; Wang et al., 2017). Theoretically, homologous chromosomes exhibit good compatibility in autopolyploids. However, in our previous study, we revealed a clear loss of chromosomal loci in the genome of autotetraploid fish (Qin et al., 2019b). In this work, we found that 4nRR possessed 200 RCC-derived chromosomes based on the results of FISH. Moreover, the two full-length transcripts demonstrated that RCC and 4nRR have different novel gene structures. Likewise, comparative analyses of AFLP fragments between RCC and 4nRR indicated that 65.40% of the bands were different. In general, these outcomes signified rapid and widespread genomic structural changes in the 4nRR genome, clearly reflecting its instability. During meiosis, Santos et al. (2003) and Corredor et al. (2007) concluded that multivalent pairing suppresses the production of diploid gametes in polyploids. While on the other hand, Parisod et al. (2010) argued that bivalent pairing is beneficial in maintaining genetic stability in polyploids. Changes in genomic can often lead to diploid-like chromosome pairing, which is a fundamental process in new polyploid formation. Since chromosomes are derived from the same genome, autopolyploids exhibit more than one potential partner in each chromosome, which might contribute to multivalent pairing during meiosis. Qin et al. (2019a) demonstrated that 4nRR shows diploid-like chromosome pairing in meiosis, which can generate normal reduced gametes. In this study, our findings revealed that obvious genetic changes can be triggered by autotetraploidization. In this respect, we therefore speculate that acute and extensive genomic structure alterations in the newly established autotetraploid genome can cause stable pairs with homologous chromosomes, which are essential to providing a physical basis for diploid-like chromosome pairing.

Albalat and Cañestro (2016) reported that genetic redundancy in polyploids can potentially facilitate adaptive divergence of duplicated genes due to genome duplication that often exhibits gene regulation. The unexpressed genes are likely to respond to gene dosage imbalances (Freeling, 2009), whereas the expressed genes may contribute to the emergence of novel functions (Magadum et al., 2013). Gene expression alterations can maintain gene balance and increase the long-term genome flexibility of autopolyploids (Higgins et al., 2012). This is especially true for some plant polyploids (Schnable et al., 2011; Buggs et al., 2012), which usually exhibit gene silencing during polyploidization. Nonetheless, full-length transcriptome dataset analysis in 4nRR has involved not only unexpression of many genes as well as the expression of a number of genes, indicating that the newly synthesized autopolyploids have undergone significant gene expression variation. We hereby hypothesize that gene expression alteration can combat gene redundancy caused by whole genome duplication in 4nRR, which promoted genome stability, and result in normal growth and development. It is important to note that the molecular functions and pathways of expressed genes might be responsible for the meiosis in 4nRR. Specifically, in this study, we observed that the expressed genes of 4nRR were enriched in meiosis I. This pathway is a cellular division required for the formation of gametes, and is essential for sexual reproduction (Webster and Schuh, 2017). In addition, the enriched KEGG pathway including proteasome, concerned with meiosis, is much associated with gametogenesis (Alleva et al., 2019). Thus, these molecular functions and pathways might also be genetic mechanisms responsible for the formation of diploid gametes in the meiosis of 4nRR.

### Epigenetic Alterations in Autotetraploid *Carassius auratus*

According to Callaway (2014), epigenetics is generally used to describe heritable stable changes in gene function that do not involve DNA sequence mutations or chromosome alterations. Interestingly, epigenetic changes have been identified to promote the survival and reproduction of polyploids and subsequently facilitate genomic changes (Li et al., 2010). Notably, DNA methylation is thought to be a widespread epigenetic phenomenon (Richards and Elgin, 2002) which is the most vital epigenetic modification associated with polyploid (Ingelbrecht et al., 1994; Xiao et al., 2013). A study by Allis and Jenuwein (2016) reported that alterations in DNA methylation induced by polyploidization regulated gene expression. In conclusion, DNA methylation change can be caused by the environmental and genomic stress such as the polyploidization process, which leads to alterations in gene expression. Previous studies have highlighted that changes in DNA methylation primarily occur in synthetic allotetraploids compared with their diploid progenitors (Fulnecek et al., 2009). In this case, MSAP data demonstrated that 4nRR exhibited 60.31% methylation pattern alterations and lower methylation levels compared to the RCC, revealing that DNA methylation alterations also occurred in the 4nRR genome. A plausible explanation for this phenomenon may be attributed to the stability of the newly established autotetraploid genome, which enables to the formation of autotetraploid lineages. For the 60.31% methylation pattern, 24.26% of the CpG loci were demethylated, while 36.05% were hypermethylated. Gene activity function is dynamically regulated by variable DNA methylation and its downstream effects (Koganti et al., 2017). In other words, many expressed genes show demethylation in the promoter region, whereas genes with a decrease or even silencing of expression exhibit promoter hypermethylation. Chen et al. (2010) argued that changes in the regulation of gene expression may underlie many phenotypic differences in the polyploids. For instance, autotetraploid rice shows larger vegetative organs, stronger stems, longer panicles and higher biomass production than diploid rice (Wu et al., 2013), which might be induced by changes in the expression patterns of genes (Li et al., 2018). In another study, the phenotypes of the 4nRR displayed apparent differences compared to RCC, such as the ratios of body length/body width, tail length/tail length, and body width/head (Qin et al., 2014), implying that DNA methylation pattern alternation is most likely to change the expression levels of the linked genes in 4nRR, which resulted in corresponding phenotypic changes.

## Data Availability Statement

The datasets presented in this study can be found in online repositories. The names of the repository/repositories and accession number(s) can be found below: https://www.ncbi.nlm.nih.gov/, SRR11585445; https://www.ncbi.nlm.nih.gov/, SRR11586700.

## Ethics Statement

The animal study was reviewed and approved by the Institute of Experimental Animals, Hunan Province, China.

## Author Contributions

SL, QQ, and CW designed the experiments. CW performed the experiments. CW and QQ performed the statistical analysis and wrote the manuscript. YwZ, HQ, CZ, LY, TY, YxZ, and TX gave final approval of the version to be published all authors read and approved the final manuscript.

## Conflict of Interest

The authors declare that the research was conducted in the absence of any commercial or financial relationships that could be construed as a potential conflict of interest.

## Footnotes

1 https://downloads.pacbcloud.com/public/software/installers/smrtlink_5.0.1.9585.zip

2 https://bigd.big.ac.cn/search?dbId=gwh&q=GWHAAIA00000000

3 https://github.com/TomSkelly/MatchAnnot

4 http://blast.ncbi.nlm.nih.gov/

5 http://www.ncbi.nlm.nih.gov/COG/

6 http://www.kegg.jp/kegg/download/

7 ftp://ftp.ncbi.nih.gov/blast/db/FASTA/nr.gz

8 http://www.uniprot.org/

9 http://www.geneontology.org/

10 https://bioinfogp.cnb.csic.es/tools/venny/index.html

## Supplementary Material

Supplementary Table 1 Novel genes in *Carassius auratus* (RCC) and autotetraploid *Carassius auratus* (4nRR).

Supplementary Table 2 AFLP and MSAP adapters and primers that were utilized in this study.
