# Supplementary material for: Genetic and Epigenetic Changes Are Rapid Responses of the Genome to the Newly Synthesized Autotetraploid Carassius auratus
Source: Front Genet. 2021 Jan 7;11:576260. doi: 10.3389/fgene.2020.576260 (PMC7817996; doi:10.3389/fgene.2020.576260)
Supplement: Supplementary Table 2 — AFLP and MSAP adapters and primers that were utilized in this study. [file Table_2.DOCX]

|  | Sequences |
| --- | --- |
| **Adaptors**  *EcoR I*-adapter a  *EcoR I*-adapter b  *Pst I*-adapter a  *Pst I*-adapter b  *Mse I*-adapter a  *Mse I*-adapter b  *Hpa II/Msp I*-adapter a  *Hpa II/Msp I*-adapter b | 5’-CTCGTAGACTGCGTACC  5’-AATTGGTACGCAGTCTAC  5’-CTCGTAGACTGCGTACATGCA  5’-TGTACGCAGTCTAC  5’-GACGATGAGTCCTGAG  5’-TACTCAGGACTCAT  5’-CGCTCAGGACTCAT  5’-GACGATGAGTCCTGAC |
| **Preselective Primers**  *EcoR I* preamplification primer  *Pst I* preamplification primer  *Mse I* preamplification primer  Hpa II/Msp I preamplification primer | 5’-GACTGCGTACCAATTCA  5’-GACTGCGTACATGCAG  5’-GATGAGTCCTGAGTAAC  5’-ATCCATGAGTCCTGCTCGG |
| **Selective Primers used in AFLP**  *Pst I-Mse I* primers  PM primer 1 5’- GACTGCGTACATGCAGAA  PM primer 2 5’- GACTGCGTACATGCAGAC  PM primer 3 5’- GACTGCGTACATGCAGAC  PM primer 4 5’- GACTGCGTACATGCAGAG  PM primer 5 5’- GACTGCGTACATGCAGAG  PM primer 6 5’- GACTGCGTACATGCAGAG  PM primer 7 5’- GACTGCGTACATGCAGAT  PM primer 8 5’- GACTGCGTACATGCAGTG | 5’-GATGAGTCCTGAGTAACTG  5’-GATGAGTCCTGAGTAACAG  5’-GATGAGTCCTGAGTAACTG  5’-GATGAGTCCTGAGTAACAC  5’-GATGAGTCCTGAGTAACAG  5’-GATGAGTCCTGAGTAACTG  5’-GATGAGTCCTGAGTAACAA  5’-GATGAGTCCTGAGTAACTG |
| **Selective Primers used in MSAP**  *EcoR I* primers  E1 5’-GACTGCGTACCAATTCACC  *Hpa II/Msp I* primers  HM1 5′-ATCCATGAGTCCTGCTCGGCTGA  HM3 5′-ATCCATGAGTCCTGCTCGGCTAT  HM5 5’-ATCCATGAGTCCTGCTCGGCTCA  HM7 5’-ATCCATGAGTCCTGCTCGGCTCC  HM9 5’-ATCCATGAGTCCTGCTCGGCTTA | E2 5’-GACTG CGTACCAATTCAGT  HM2 5’-ATCCATGAGTCCTGCTCGGCTGT  HM4 5’-ATCCATGAGTCCTGCTCGGCTAC  HM6 5’-ATCCATGAGTCCTGCTCGGCTCT  HM8 5’-ATCCATGAGTCCTGCTCGGCTTC  HM10 5’-ATCCATGAGTCCTGCTCGGCTTT |
